# Multistate markov model analysis of metabolic syndrome progression in a community-based longitudinal cohort study

**DOI:** 10.1186/s13098-026-02146-8

**Published:** 2026-04-28

**Authors:** Hung-pin Chen, Amy Ming-Fang Yen, Yen-Po Yeh, Dih-Ling Luh

**Affiliations:** 1 Department of Public Health, Chung Shan Medical University, Taichung, Taiwan; 2 Changhua Public Health Bureau, Changhua, Taiwan; 3 College of Oral Medicine, Taipei Medical University, Taipei, Taiwan; 4 Graduate Institute of Epidemiology and Preventive Medicine, College of Public Health, National Taiwan University, Taipei, Taiwan; 5 Department of Family and Community Medicine, Chung Shan Medical University Hospital, Taichung, Taiwan

**Keywords:** Metabolic syndrome, Multistate Markov model, Component-specific progression, Sex effects, Age effects, Community-based screening

## Abstract

**Background:**

Metabolic syndrome (MetS) is a cluster of metabolic abnormalities that are strongly associated with cardiovascular disease and diabetes. However, there is limited evidence on how MetS develops from individual metabolic abnormalities. This study investigated which initial metabolic components accelerate the progression of MetS and analyzed how sex, age, and residential location affect these developmental trajectories.

**Methods:**

This study analyzed screening data from Changhua County (2005–2021) to conduct a retrospective generational study of 41,109 adults aged ≥ 30 years and 63,114 records. Metabolic abnormalities were categorized into four states (state 0–3), and transition rates were estimated using continuous-time multistate Markov models and analyzed based on initial abnormality type, sex, and age. Progression was defined as a continuous transition across the metabolic states from State 0 to 1, 1 to 2, and ultimately to State 3, reflecting the accumulation of metabolic abnormalities over time.

**Results:**

Triglyceride (TG) and waist circumference abnormalities exhibited the most rapid progression intensities (0.344 and 0.326/year, respectively). Crucially, the mean sojourn time—the expected duration individuals remained in each state before transitioning—was estimated at 3.07 years for State 1 with one abnormality and 2.79 years for State 2 with two abnormalities. The analysis revealed that women accumulated metabolic risk at a faster rate than men after midlife, while rural residents exhibited accelerated deterioration, specifically driven by their higher TG and WC levels compared to urban residents.

**Conclusion:**

MetS progression is rapid but reversible during the early stages. The short mean sojourn times warrant a recommendation for follow-up screening intervals of 2–3 years for individuals who present with one or more metabolic abnormalities, as this timeframe captures the window of opportunity for reversal. Public health interventions should prioritize lipid management and weight control, particularly targeting rural populations and postmenopausal women, who exhibit the steepest disease progression.

**Supplementary Information:**

The online version contains supplementary material available at 10.1186/s13098-026-02146-8.

## Introduction

Metabolic syndrome (MetS) is a cluster of metabolic abnormalities that increase the risk of type 2 diabetes, cardiovascular disease, and shortened lifespan. The prevalence of MetS varies considerably across the Asia-Pacific region, ranging from 11.9% to 49.0%, while the global prevalence remains between 20% and 25%, primarily due to rising obesity rates and an aging population [4, 17, 21]. According to the NCEP ATP III criteria, MetS is defined by abnormalities in at least three of five indicators: blood pressure, fasting blood glucose, waist circumference, triglyceride (TG) levels, and high-density lipoprotein cholesterol (HDL [2, 19].

Although the clinical diagnosis of MetS is binary, its pathophysiology is a gradual, dynamic process involving a sequence of metabolic disturbances. The likelihood of early onset and trajectory of subsequent progression varies significantly across individuals, depending on their distinct combinations of risk components [9, 18].

Previous research has limitations. Although studies such as Hwang et al. [9] have utilized Markov models to investigate the effects of initial components, their focus on a younger population (mean age 32 years) limits the generalizability of their findings to aging cohorts, where metabolic risks accelerate. Furthermore, few studies have leveraged such a large-scale dataset (*N* = 41,109) with an extended follow-up period (16 years) to deconstruct these trajectories. While recent systematic reviews have confirmed the rising global burden of MetS [16, 17], evidence regarding the dynamic evolution from specific initial abnormalities remains limited in older community populations.

Crucially, the burden of MetS is not uniformly distributed across populations. Recent epidemiological evidence has highlighted significant disparities in the prevalence of MetS associated with demographic and environmental determinants. For instance, age-related metabolic decline [7, 16], sex-specific hormonal variations, particularly concerning menopause [11], and lifestyle disparities between urban and rural settings [10, 16] influence metabolic risk profiles. However, most contemporary research has focused on static prevalence rates rather than dynamic ones. Whether these factors actively accelerate the rate of disease progression or hinder recovery remains unknown. Therefore, stratified analysis is essential to characterize dynamic transition patterns across different subpopulations and facilitate the development of precision public health strategies.

To bridge these gaps, this study analyzed 16 years of community screening data (2005–2021) from a large cohort in central Taiwan (*N* = 41,109). We applied a continuous-time multistate Markov model —a method increasingly applied in chronic disease epidemiology to analyze the formation and reversal of metabolic risk factors [14] —to achieve three main objectives: (1) to estimate the bidirectional transition intensities between metabolic states (progression and regression); (2) to evaluate how specific initial abnormal components influence the speed of progression and regression ; and (3) to assess the effects of sex, age, and residential setting on these metabolic trajectories. By elucidating the temporal evolution of MetS, we aimed to provide evidence-based recommendations for targeted prevention strategies.

## Materials and methods

This retrospective, community-based cohort study was based on data from the “Integrated Community Screening Program” conducted in Changhua County between 2005 and 2021. This program collected health examination records from residents aged 30 years and above. The participants were community volunteers who participated in locally organized health screening activities.

**Fig. 1:**
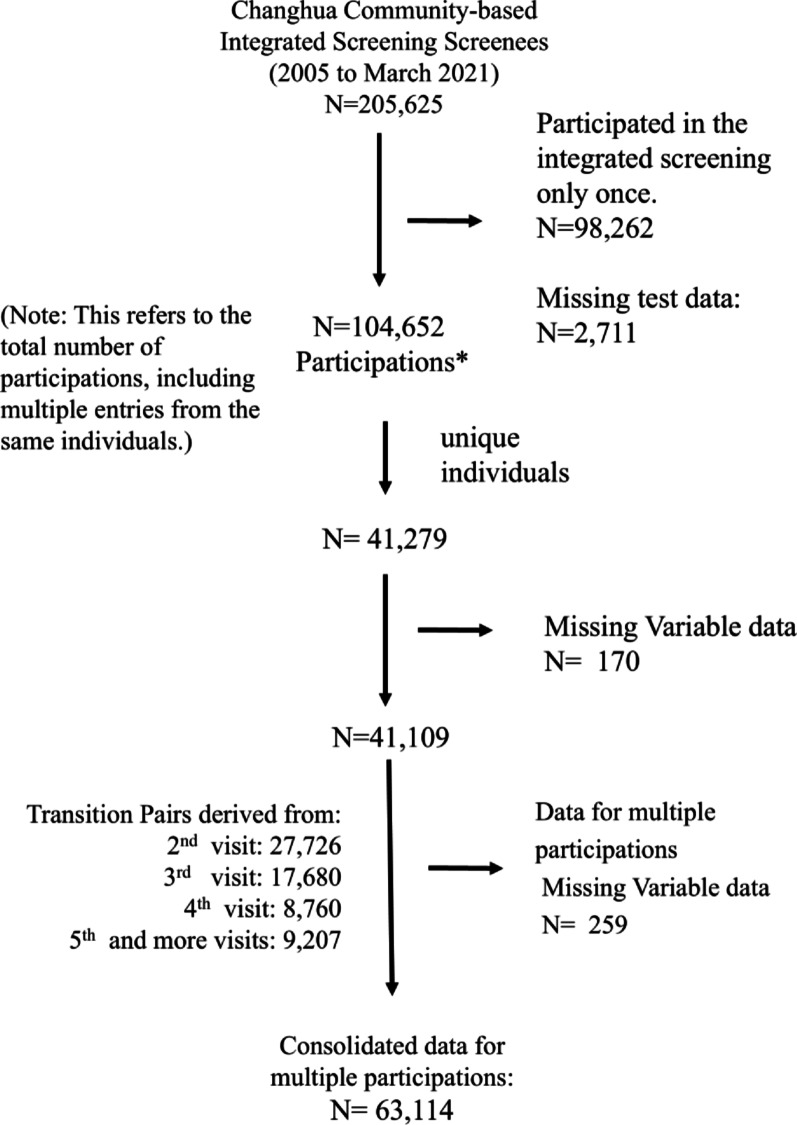
Flowchart of Participant Selection and Data Consolidation. The flow diagram illustrates the process of identifying unique participants (*N* = 41,109) from the Changhua Community-Based Integrated Screening Program (2005–2021). The lower section details the generation of transition pairs (*N* = 63,114) derived from follow-up visits (2nd, 3rd, 4th, and ≥ 5th visits), which served as the unit of analysis for the continuous-time Markov model

A total of 205,625 participants were initially enrolled in the study. Individuals who had only one screening, lacked complete personal identification numbers (PINs), or had missing MetS-related data were excluded from the study. After excluding 98,262 participants who underwent only one screening and 2,711 with missing data,104,652 participants remained, and 63,114 valid examination records were obtained for analysis (Fig. 1).

### MetS component definitions

The components followed the NCEP ATP III and Taiwan National Health Service (NHS) guidelines as follows:

- Elevated blood pressure (BP): systolic BP ≥ 130 mmHg, diastolic BP ≥ 85 mmHg, or self-reported hypertension.
- Elevated fasting plasma glucose (FPG) levels: ≥ 100 mg/dL or self-reported diabetes.
- Abdominal obesity (waist circumference, WC): ≥ 90 cm (men) or ≥ 80 cm (women).
- Elevated triglyceride (TGs) levels: ≥ 150 mg/dL.
- Low HDL-C: < 40 mg/dL (men) or < 50 mg/dL (women).

The five MetS components included fasting plasma glucose (FPG), and impaired fasting glucose (IFG) was treated as one of the initial abnormality types in the multistate model.

Biochemical indicators, including TG, HDL-C, and FPG levels, were measured in fasting venous blood samples using a certified clinical analyzer. Waist circumference and blood pressure were measured by trained nurses according to standardized protocols. Metabolic syndrome was diagnosed when three or more of the five indicators were abnormal.

### State construction

At each screening, the participants were categorized into four sequential states based on the number of abnormal components: State 0 (no abnormalities), State 1 (one abnormality), State 2 (two abnormalities), and State 3 (three to five abnormalities; metabolic syndrome). The structure of the model and the allowed transitions are presented in Supplementary Fig. 1.

We assumed a stepwise progression and regression structure (State 0 **↔** 1**↔** 2 **↔** 3). In clinical practice, individuals may accumulate two or more metabolic abnormalities within the same screening interval, especially when examinations are performed several years apart. Within the continuous-time Markov framework, such within-interval accumulation is represented as a rapid sequence of intermediate transitions (e.g., 0 → 1 → 2), rather than a single instantaneous jump between non-adjacent states. Mathematically, the probability of two distinct changes occurring at exactly the same instant is considered negligible; however, the model permits multiple transitions to occur over the calendar periods between visits. This stepwise specification provides a parsimonious description of the dynamic process while enabling stable estimation of transition intensities and mean sojourn times.

In this study, disease progression was defined as forward transitions between metabolic states (State 0→1, 1→2, and 2→3), reflecting the cumulative accumulation of metabolic abnormalities. Reverse transitions (State 1→0, 2→1, and 3→2) were interpreted as metabolic recovery. In addition, post-baseline state distributions (State 0–3) following entry into State 1 were characterized by changes in state occupancy probabilities over time.

### Covariates

The control variables in this study included demographic characteristics (sex, age, and education level), lifestyle factors (smoking status, alcohol consumption, and physical activity), and dietary and health indicators (intake of vegetables, fruits, and sugary foods and medical history). All variables were self-reported and analyzed in conjunction with the screening data.

### Bias

The research team discovered multiple potential sources of bias during their investigation. Participants who chose to join the screening might show different results than other participants because of healthy volunteer bias, while their self-reported lifestyle information could be influenced by recall errors and social desirability bias. Those who received multiple screenings showed different characteristics than those who received only one screening, resulting in follow-up bias. Standardized measurement methods were used, and all available repeated records were included to reduce both selection and misclassification errors.

### Statistics analysis

Descriptive statistics were used to examine the baseline distribution patterns across the different metabolic states, and chi-square tests were conducted to examine group differences. The state transition rates (h1-h6) were estimated by constructing a continuous-time multistate Markov model with a generator matrix Q, and the state transition probability matrix P(t) was obtained through eigenvalue decomposition.

The expression for P(t) is as follows: P(t) = A × exp (D × t) × A⁻¹, where A represents the eigenvector matrix, D represents the eigenvalue matrix, and *t* denotes the exact time interval between consecutive observations for each individual.

Model parameters were estimated using the maximum likelihood estimation (MLE) method, employing the NLPNRA algorithm in SAS/IML, and referencing the SAS macro set described by Wu et al. [8].

Missing data were handled using a complete case analysis. As missing values accounted for less than 5% of the total dataset, multiple imputations were not performed.

The transition intensities of different initial anomaly types (BP, FPG, WC, TG, and HDL) in each progression stage (0→1, 1→2, and 2→3) were compared, and statistical significance was evaluated using a Z-test. Furthermore, the transition pathways and cumulative risk patterns over a 16-year period starting from the State 1 were simulated. All statistical analyses and visualizations were performed using SAS version 9.4 (SAS Institute, Inc., Cary, NC, USA).

## Results

### Distribution of sample characteristics and metabolic abnormalities

A total of 41,109 participants were included in the analysis (Table 1), contributing to 63,114 screening records. The mean age of the participants was 56.9 ± 9.7 years old. The median follow-up interval between consecutive screening visits was 3.07 years (interquartile range [IQR]: 2.78–4.87 years), providing a sufficient observation window to detect the metabolic state transitions. The analysis in Table 2 demonstrates that metabolic abnormalities were significantly associated with personal characteristics and life factors, existing medical conditions, and biochemical indicators (*p* < 0.0001). The prevalence of three to five metabolic abnormalities was higher among men, older adults, individuals with lower levels of education, and rural residents. Individuals who smoke regularly, consume alcohol frequently, and engage in insufficient physical activity tend to develop a greater number of metabolic abnormalities. The study found that MetS was most prevalent among individuals with diabetes, chronic kidney disease, hyperlipidemia, and those aged ≥ 40 years with elevated GOT or GPT levels, as 47% of these patients fulfilled MetS criteria. Individuals who regularly consume sugary drinks and snacks have an elevated risk of developing various metabolic disorders.

**Table 1:** Baseline characteristics of individual-level factors by mets component numbers (unique individuals)

			MetS component number								
			0		1		2		3–5		
Variable	N	%	N	%	N	%	N	%	N	%	p value
**Participants**	41,109	100	9617	23.4	11,236	27.3	9239	22.5	11,017	26.8	
**Demographic variables**											
**Gender**											
Men	14,283	35.1	2681	18.8	3908	27.4	3553	24.9	4141	29.0	< 0.0001
Female	26,826	65.9	6936	25.9	7328	27.3	5686	21.2	6876	25.6	
**Age**											
<50	16,612	40.8	5512	33.2	4894	29.5	3114	18.7	3092	18.6	< 0.0001
50–64	20,384	50.1	3629	17.8	5356	26.3	5001	24.5	6398	31.4	
≥65	4113	10.1	476	11.6	986	24.0	1124	27.3	1527	37.1	
**Education**											
≤ Middle school	22,650	55.6	4204	18.6	5791	25.6	5490	24.2	7165	31.6	< 0.0001
High school	11,665	28.6	3260	27.9	3410	29.2	2439	20.9	2556	21.9	
≥ College	6794	16.7	2153	31.7	2035	30.0	1310	19.3	1296	19.1	
**Lifestyle habits**											
**Smoke**											
None	32,886	80.8	8074	24.6	9060	27.5	7258	22.1	8494	25.8	< 0.0001
Quit	3289	8.1	555	16.9	871	26.5	821	25.0	1042	31.7	
Current smoker	4934	12.1	988	20.0	1305	26.4	1160	23.5	1481	30.0	
**Alcohol**											
None	37,351	91.7	8987	24.1	10,271	27.5	8252	22.1	9841	26.3	< 0.0001
Quit	720	1.8	137	19.0	167	23.2	169	23.5	247	34.3	
Current drinker	3038	7.5	493	16.2	798	26.3	818	26.9	929	30.6	
**Exercise**											
No	17,143	42.1	4108	24.0	4591	26.8	3875	22.6	4569	26.7	< 0.0001
< 150 min/wk	14,466	35.5	3660	25.3	4157	28.7	3054	21.1	3595	24.9	
≥ 150 min/wk	9500	23.3	1849	19.5	2488	26.2	2310	24.3	2853	30.0	
**Chronic disease**											
DM											
No	39,154	96.2	9617	24.6	11,052	28.2	8795	22.5	9690	24.7	< 0.0001
Yes	1955	4.8	0	0.0	184	9.4	444	22.7	1327	67.9	
Hypertension											
No	35,801	87.9	9617	26.9	10,435	29.1	7832	21.9	7917	22.1	< 0.0001
Yes	5308	13.0	0	0.0	801	15.1	1407	26.5	3100	58.4	
Hyperlipidemia											
No	39,705	97.5	9495	23.9	10,996	27.7	8910	22.4	10,304	26.0	< 0.0001
Yes	1404	3.4	122	8.7	240	17.1	329	23.4	713	50.8	
CKD											
No	40,842	100.3	9575	23.4	11,171	27.4	9177	22.5	10,919	26.7	< 0.0001
Yes	267	0.7	42	15.7	65	24.3	62	23.2	98	36.7	
**Health status**											
**GOT**											
≦ 40	39,081	96.0	9380	24.0	10,884	27.8	8795	22.5	10,022	25.6	< 0.0001
> 40	2028	5.0	237	11.7	352	17.4	444	21.9	995	49.1	
**GPT**											
≦ 40	37,231	91.4	9240	24.8	10,598	28.5	8375	22.5	9018	24.2	< 0.0001
> 40	3878	9.5	377	9.7	638	16.5	864	22.3	1999	51.5	
**Diet habit**											
Vegetables ≧ 3 bowls/day											
No	35,908	88.2	8452	23.5	9806	27.3	8073	22.5	9577	26.7	0.2277
Yes	5201	12.8	1165	22.4	1430	27.5	1166	22.4	1440	27.7	
Fruit ≧ 7 times/week											
No	24,945	61.3	6083	24.4	6828	27.4	5521	22.1	6513	26.1	< 0.0001
Yes	16,164	39.7	3534	21.9	4408	27.3	3718	23.0	4504	27.9	
Little beverage											
No	12,998	31.9	3257	25.1	3683	28.3	2792	21.5	3266	25.1	< 0.0001
Yes	28,111	69.0	6360	22.6	7553	26.9	6447	22.9	7751	27.6	
Little snack											
No	19,161	47.1	5239	27.3	5467	7.0	3993	20.8	4462	23.3	< 0.0001
Yes	21,948	53.9	4378	19.9	5769	26.3	5246	23.9	6555	29.9	
**Area**											
City	7826	18.0	2249	28.7	2197	28.1	1603	20.5	1777	22.7	< 0.0001
Town	9721	22.3	2256	23.2	2753	28.3	2191	22.5	2521	25.9	
Township	23,562	54.1	5112	21.7	6286	26.7	5445	23.1	6719	28.5	

The table shows the frequency (N, %) of participants across MetS component numbers (0, 1, 2, and 3–5 risk factors). MetS numbers indicate the count of risk factors, where 0 = none, 1 = one, 2 = two, and 3–5 = three or more. N and % represent the count and percentage of participants, and p values show statistical significance (*p* < 0.05). Education, Lifestyle Habits, DM, Hypertension, Diet Habit categories are based on self-reports

**Table 2:** Pooled distribution of all screening records by metabolic syndrome (mets) component numbers (multiple records per individual)

			MetS component number								
	Total		0		1		2		3–5		
Variable	N	%	N	%	N	%	N	%	N	%	p value
Participants	63,114	100	14,519	23.0	17,327	27.5	14,374	22.8	16,894	26.8	
Demographic variables											
**Gender**											
Men	22,331	51.3	4103	18.4	6118	27.4	5629	25.2	6481	29.0	< 0.0001
Female	40,783	93.7	10,416	25.5	11,209	27.5	8745	21.4	10,413	25.5	
**Age**											
<50	23,876	54.8	7986	33.4	7106	29.8	4447	18.6	4337	18.2	< 0.0001
50–64	30,424	69.9	5543	18.2	8105	26.6	7508	24.7	9268	30.5	
≥65	8814	20.2	990	11.2	2116	24.0	2419	27.4	3289	37.3	
**Education**											
≤ Middle school	35,126	80.7	6473	18.4	9042	25.7	8570	24.4	11,041	31.4	< 0.0001
High school	17,657	40.6	4892	27.7	5196	29.4	3726	21.1	3843	21.8	
≥ college	10,331	23.7	3154	30.5	3089	29.9	2078	20.1	2010	19.5	
**Lifestyle habits**											
**Smoke**											
None	51,112	117.4	12,288	24.0	14,170	27.7	11,471	22.4	13,183	25.8	< 0.0001
Quit	5181	11.9	833	16.1	1365	26.3	1293	25.0	1690	32.6	
Current smoker	6821	15.7	1398	20.5	1792	26.3	1610	23.6	2021	29.6	
**Alcohol**											
None	57,517	132.1	13,587	23.6	15,909	27.7	12,892	22.4	15,129	26.3	< 0.0001
Quit	1091	2.5	192	17.6	255	23.4	265	24.3	379	34.7	
Current drinker	4506	10.4	740	16.4	1163	25.8	1217	27.0	1386	30.8	
**Exercise**											
No	24,688	56.7	5966	24.2	6610	26.8	5584	22.6	6528	26.4	< 0.0001
< 150 min/week	22,006	50.6	5493	25.0	6375	29.0	4690	21.3	5448	24.8	
≥ 150 min/week	16,420	37.7	3060	18.6	4342	26.4	4100	25.0	4918	30.0	
**Chronic disease**											
DM											
No	60,011	137.9	14,519	24.2	17,021	28.4	13,667	22.8	14,804	24.7	< 0.0001
Yes	3103	7.1		0.0	306	9.9	707	22.8	2090	67.4	
Hypertension											
No	54,427	125.0	14,519	26.7	15,990	29.4	12,014	22.1	11,904	21.9	< 0.0001
Yes	8687	20.0		0.0	1337	15.4	2360	27.2	4990	57.4	
Hyperlipidemia											
No	60,777	139.6	14,305	23.5	16,912	27.8	13,801	22.7	15,759	25.9	< 0.0001
Yes	2337	5.4	214	9.2	415	17.8	573	24.5	1135	48.6	
CKD											
No	62,661	143.9	14,453	23.1	17,220	27.5	14,268	22.8	16,720	26.7	< 0.0001
Yes	453	1.0	66	14.6	107	23.6	106	23.4	174	38.4	
**Health status**											
**GOT**											
≤ 40	60,075	138.0	14,152	23.6	16,812	28.0	13,697	22.8	15,414	25.7	< 0.0001
> 40	3039	7.0	367	12.1	515	16.9	677	22.3	1480	48.7	
**GPT**											
≤ 40	57,384	131.8	13,949	24.3	16,388	28.6	13,093	22.8	13,954	24.3	< 0.0001
> 40	5730	13.2	570	9.9	939	16.4	1281	22.4	2940	51.3	
**Diet habbit**											
Vegetables ≥ 3 bowls/day											
No	54,622	125.5	12,666	23.2	14,983	27.4	12,415	22.7	14,558	26.7	0.6398
Yes	8492	19.5	1853	21.8	2344	27.6	1959	23.1	2336	27.5	
Fruit ≥ 7 times/week											
No	37,175	85.4	8987	24.2	10,185	27.4	8266	22.2	9737	26.2	< 0.0001
Yes	25,939	59.6	5532	21.3	7142	27.5	6108	23.5	7157	27.6	
Little beverage											
No	18,709	43.0	4674	25.0	5308	28.4	4017	21.5	4710	25.2	< 0.0001
Yes	44,405	102.0	9845	22.2	12,019	27.1	10,357	23.3	12,184	27.4	
Little snack											
No	28,921	66.4	7814	27.0	8269	28.6	6091	21.1	6747	23.3	< 0.0001
Yes	34,193	78.5	6705	19.6	9058	26.5	8283	24.2	10,147	29.7	
**Area**											
City	11,272	25.9	3173	28.1	3171	28.1	2401	21.3	2527	22.4	< 0.0001
Town	14,265	32.8	3317	23.3	4128	28.9	3251	22.8	3569	25.0	
Township	37,577	86.3	8029	21.4	10,028	26.7	8722	23.2	10,798	28.7	

The frequency distribution (N, %) of event-level factors influencing MetS component numbers, where individuals may have contributed multiple records. MetS component numbers represent the count of risk factors, with 0 = no factors, 1 = one factor, 2 = two factors, and 3–5 = three or more. N and % indicate the number and percentage of records within each category. p values represent the statistical significance of differences across MetS component numbers, with values < 0.05 considered significant

The data in Tables 3, 4 and 5reveal that hypertension occurs most frequently among people with one metabolic disorder at 29.2%, followed by increased waist circumference at 21.4% and low HDL-C at 20.1%. Hypertension was most common among male participants, whereas the highest proportion of female participants had both hypertension and low HDL-C levels. Hypertension and elevated fasting blood glucose levels were most common among older participants, whereas low HDL-C levels were more frequently observed among younger participants. Participants who engaged in regular exercise, ate fruits and vegetables daily, and limited their intake of sugary drinks exhibited lower blood pressure levels and smaller waist circumferences.

**Table 3:** Distribution of demographic and lifestyle characteristics at the time of initial detection of risk factors

			MetS component										
	Total		BP		IFG		Waist		TG		HDL		
Variable	N	%	N	%	N	%	N	%	N	%	N	%	p value
Participants	105,484	100	30,782	29.2	16,571	15.7	22,570	21.4	14,410	13.7	21,151	20.1	
Demographic variables													
**Gender**													
Men	39,951	37.9	13,143	32.9	6506	16.3	7110	17.8	6806	17.0	6386	16.0	< 0.0001
Female	65,533	62.1	17,639	26.9	10,065	15.4	15,460	23.6	7604	11.6	14,765	22.5	
**Age**													
<50	30,876	29.3	8157	26.4	3686	11.9	6568	21.3	4529	14.7	7936	25.7	< 0.0001
50–64	55,938	53.0	16,662	29.8	9280	16.6	11,912	21.3	7919	14.2	10,165	18.2	
≥65	18,670	17.7	5963	31.9	3605	19.3	4090	21.9	1962	10.5	3050	16.3	
**Education**													
≤ Middle school	65,303	61.9	18,734	28.7	11,172	17.1	14,733	22.6	8409	12.9	12,255	18.8	< 0.0001
High school	26,010	24.7	7779	29.9	3567	13.7	5188	19.9	3817	14.7	5659	21.8	
≥ College	14,171	13.4	4269	30.1	1832	12.9	2649	18.7	2184	15.4	3237	22.8	
**Lifestyle habits**													
**Smoke**													
None	83,446	79.1	24,133	28.9	13,232	15.9	18,445	22.1	10,255	12.3	17,381	20.8	< 0.0001
Quit	9910	9.4	3261	32.9	1665	16.8	1908	19.3	1616	16.3	1460	14.7	
Current smoker	12,128	11.5	3388	27.9	1674	13.8	2217	18.3	2539	20.9	2310	19.0	
**Alcohol**													
None	94,913	90.0	27,403	28.9	14,907	15.7	20,597	21.7	12,323	13.0	19,683	20.7	< 0.0001
Quit	2149	2.0	622	28.9	363	16.9	396	18.4	352	16.4	416	19.4	
Current drinker	8422	8.0	2757	32.7	1301	15.4	1577	18.7	1735	20.6	1052	12.5	
**Exercise**													
No	40,760	38.6	11,295	27.7	6165	15.1	9079	22.3	5751	14.1	8470	20.8	< 0.0001
< 150 min/wk	34,862	33.0	10,275	29.5	5041	14.5	7217	20.7	4965	14.2	7364	21.1	
≥ 150 min/wk	29,862	28.3	9212	30.8	5365	18.0	6274	21.0	3694	12.4	5317	17.8	

The table shows the frequency distribution (N, %) of participants with one or more MetS risk factors, stratified by demographic and lifestyle characteristics. The column headers represent the specific initial risk factor: elevated blood pressure (BP), impaired fasting glucose (IFG), increased waist circumference (Waist), high triglycerides (TG), and low high-density lipoprotein (HDL). Chi-square tests were used to assess group differences; p values < 0.05 indicate statistical significance

**Table 4:** Distribution of clinical, biochemical, and dietary characteristics at the time of initial detection of risk factors

			MetS component										
	Total		BP		IFG		Waist		TG		HDL		
Variable	N	%	N	%	N	%	N	%	N	%	N	%	p value
Participants	105,484	100	30,782	29.2	16,571	15.7	22,570	21.4	14,410	13.7	21,151	20.1	
**Chronic disease**													
DM													
No	95,930	90.9	28,633	29.8	13,468	14.0	20,757	21.6	13,349	13.9	19,723	20.6	< 0.0001
Yes	9554	9.1	2149	22.5	3103	32.5	1813	19.0	1061	11.1	1428	14.9	
Hypertension													
No	81,211	77.0	22,095	27.2	12,746	15.7	17,488	21.5	11,388	14.0	17,494	21.5	< 0.0001
Yes	24,273	23.0	8687	35.8	3825	15.8	5082	20.9	3022	12.5	3657	15.1	
Hyperlipidemia													
No	99,771	94.6	29,257	29.3	15,594	15.6	21,363	21.4	13,339	13.4	20,218	20.3	< 0.0001
Yes	5713	5.4	1525	26.7	977	17.1	1207	21.1	1071	18.7	933	16.3	
CKD													
No	104,556	99.1	30,502	29.2	16,412	15.7	22,368	21.4	14,283	13.7	20,991	20.1	0.2606
Yes	928	0.9	280	30.2	159	17.1	202	21.8	127	13.7	160	17.2	
**Health status**													
**GOT**													
≤ 40	98,144	93.0	28,842	29.4	15,260	15.5	20,883	21.3	13,295	13.5	19,864	20.2	< 0.0001
> 40	7340	7.0	1940	26.4	1311	17.9	1687	23.0	1115	15.2	1287	17.5	
**GPT**													
≤ 40	91,231	86.5	27,085	29.7	14,162	15.5	19,289	21.1	12,032	13.2	18,663	20.5	< 0.0001
> 40	14,253	13.5	3697	25.9	2409	16.9	3281	23.0	2378	16.7	2488	17.5	
**Diet habbit**													
Vegetables ≥ 3 bowls/day													
No	90,930	86.2	26,496	29.1	14,213	15.6	19,416	21.4	12,499	13.7	18,306	20.1	0.0558
Yes	14,554	13.8	4286	29.4	2358	16.2	3154	21.7	1911	13.1	2845	19.5	
Fruit ≥ 7 times/week	0												
No	60,906	57.7	17,597	28.9	9428	15.5	12,946	21.3	8552	14.0	12,383	20.3	< 0.0001
Yes	44,578	42.3	13,185	29.6	7143	16.0	9624	21.6	5858	13.1	8768	19.7	
Little beverage													
No	29,841	28.3	8365	28.0	4225	14.2	6606	22.1	4412	14.8	6233	20.9	< 0.0001
Yes	75,643	71.7	22,417	29.6	12,346	16.3	15,964	21.1	9998	13.2	14,918	19.7	
Little snack													
No	44,021	41.7	12,769	29.0	6525	14.8	9308	21.1	5975	13.6	9444	21.5	< 0.0001
Yes	61,463	58.3	18,013	29.3	10,046	16.3	13,262	21.6	8435	13.7	11,707	19.0	
**Area**													
City	16,785	15.9	5013	29.9	2404	14.3	3171	18.9	2431	14.5	3766	22.4	< 0.0001
Town	23,199	22.0	6823	29.4	3650	15.7	4757	20.5	3223	13.9	4746	20.5	
Township	65,500	62.1	18,946	28.9	10,517	16.1	14,642	22.4	8756	13.4	12,639	19.3	

The table shows the frequency distribution (N, %) of participants with one or more MetS risk factors, stratified by **health status**,** chronic diseases**,** dietary habits**,** and residential area**. The column headers represent the specific initial risk factor: elevated blood pressure (BP), impaired fasting glucose (IFG), increased waist circumference (Waist), high triglycerides (TG), and low high-density lipoprotein (HDL). Chi-square tests were used to assess group differences; p values < 0.05 indicate statistical significance

**Table 5:** Transition rates of MetS indicators compared to the base transition rate

		Transition rate												
		0→1			1→2		2→3–5		0←1		1←2		2←3–5	
Variable	Total N	Rate	*p*		Rate	*p*	Rate	*p*	Rate	*p*	Rate	*p*	Rate	*p*
**Base Transition Rate**	63,114	0.201	-		0.223	-	0.198	-	0.111	-	0.181	-	0.113	-
		**Risk factor subgroups**		**Total** **N**										
		**BP**		30,782	0.202	0.311	0.175	0.274	0.162	0.542	0.180	0.956	0.110	0.892
		**IFG**		16,571	0.224	0.239	0.193	0.533	0.110	0.743	0.186	0.621	0.095	0.843
		**Waist**		22,570	0.326	< 0.001	0.207	0.042	0.171	0.621	0.185	0.912	0.103	0.839
		**TG**		14,410	0.344	< 0.001	0.216	0.048	0.207	0.032	0.253	0.013	0.108	0.804
		**HDL**		21,151	0.210	0.732	0.187	0.736	0.094	0.620	0.212	0.377	0.105	0.767

This table compares transition rates of individual MetS components—blood pressure (BP), impaired fasting glucose (IFG), waist circumference (Waist), triglycerides (TG), and high-density lipoprotein (HDL)—against the base rate. Forward transitions (0→1, 1→2, 2→3–5) indicate progression, while reverse transitions (0←1, 1←2, 2←3–5) indicate regression

P values were calculated using two-proportion Z tests. The pooled proportion was used to estimate standard error. Significant differences from the base rate are indicated by *p* < 0.05

### Rates of transition between metabolic states in the community (objective 1)

This study employed a continuous-time multistate Markov model to estimate the yearly transition rates between State 0 and 3. Within the overall sample, the instantaneous progression intensities from State 0→1 (healthy→one abnormality) were 0.201/year for State 0→1, 0.223/year for State 1→2, and 0.198/year for State 2 →3 (progression to MetS). The reverse (recovery) intensities were lower: 0.111/year for State 1→0, 0.181/year for State 2→1, and 0.113/year for State 3→2. The detailed matrix of observed transitions (raw counts) and estimated annual transition intensities are provided in Supplementary Table 1. Based on the model estimates, the mean sojourn time (expected duration of stay) was calculated for each metabolic state. Participants remained in State 1 (single risk factor) for an average of 3.07 years and in State 2 (two risk factors) for 2.79 years before transitioning to a different state. This study shows that metabolic problems progress rapidly but require prolonged recovery periods, leading to the accumulation of various health risks.

### Initial abnormality and subsequent transition dynamics (study objective 2)

The first abnormal element (initiator) demonstrated that the forward transition intensities for TG reached their peak at 0.344/year for State 1→2 (*p* < 0.001) and 0.216/year for State 2→3 (*p* = 0.048). The reverse (recovery) transitions were also elevated at 0.207/year for State 1→0 (*p* = 0.032) and 0.253/year for State 2→1 (*p* = 0.013). Research shows that TG abnormalities lead to high metabolic instability because their progression and regression patterns do not follow standard baseline rates. The progression of abdominal obesity exceeded other risk factors, advancing at a rate of 0.326/year from State 1 to State 2 (*p* < 0.001) and 0.207/year from State 2 to State 3 (*p* = 0.042). In contrast, the reverse intensities were not significantly different from the overall averages, and the results were not statistically significant. Blood pressure and fasting plasma glucose abnormalities revealed that forward and reverse transition intensities were similar to the cohort averages (all *p* > 0.20). HDL-C initiators displayed the lowest forward and reverse transition intensities among all groups, with values that did not differ from the total group averages.

### Differences by sex, age, and place of residence (objective 3)

**Fig. 2:**
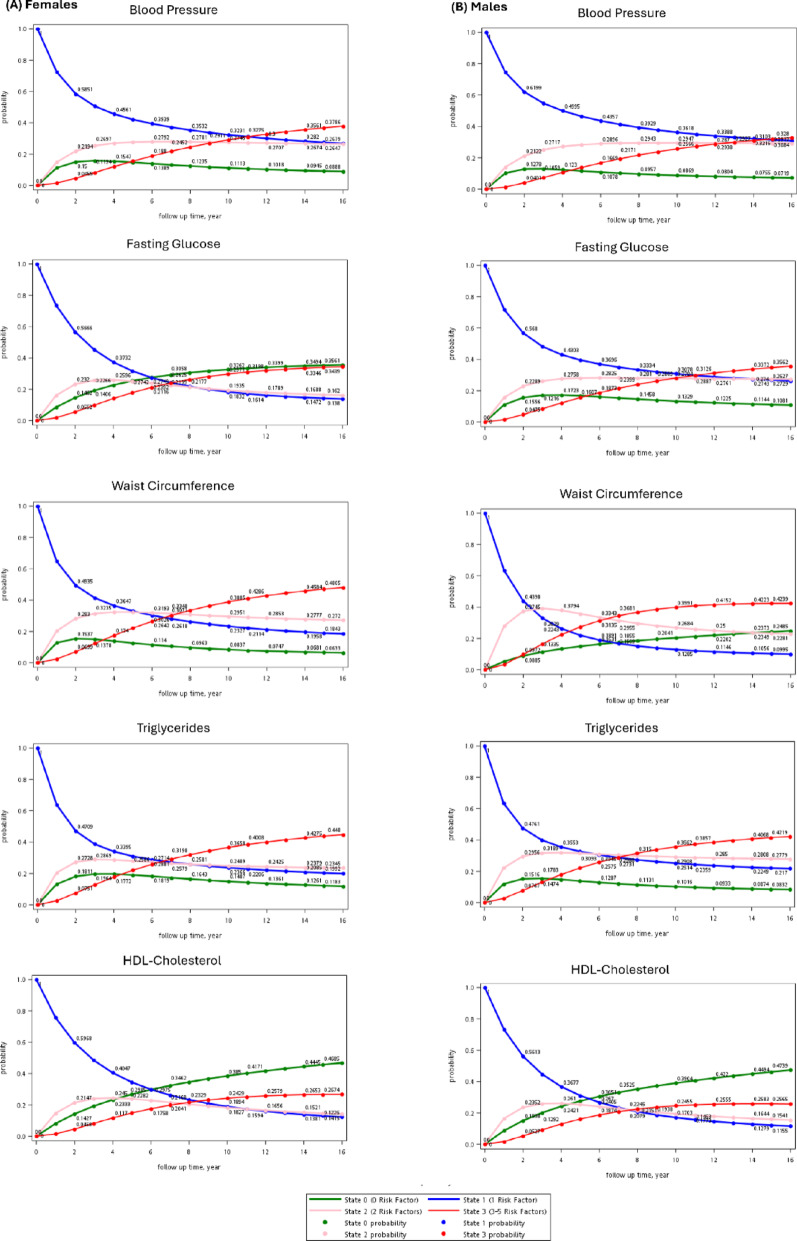
Consolidated dynamic transition probabilities from State 1 (single risk factor) over a 16-year follow-up, stratified by sex. The panels display the probability trajectories starting from five specific initial metabolic components: Blood Pressure (BP), Fasting Glucose (IFG), Waist Circumference, Triglycerides (TG), and HDL-C. (**A**) Females. (**B**) Males. The curves represent the probability of occupancy in each state over time: the **blue line** indicates remaining in the initial state (State 1); the **green line** indicates recovery to a risk-free state (State 0); the **pink line** indicates progression to two risk factors (State 2); and the **red line** indicates progression to Metabolic Syndrome (State 3, ≥ 3 risk factors). Note the varying rates of progression to State 3 (**red line**) depending on the initial component and sex

**Fig. 3:**
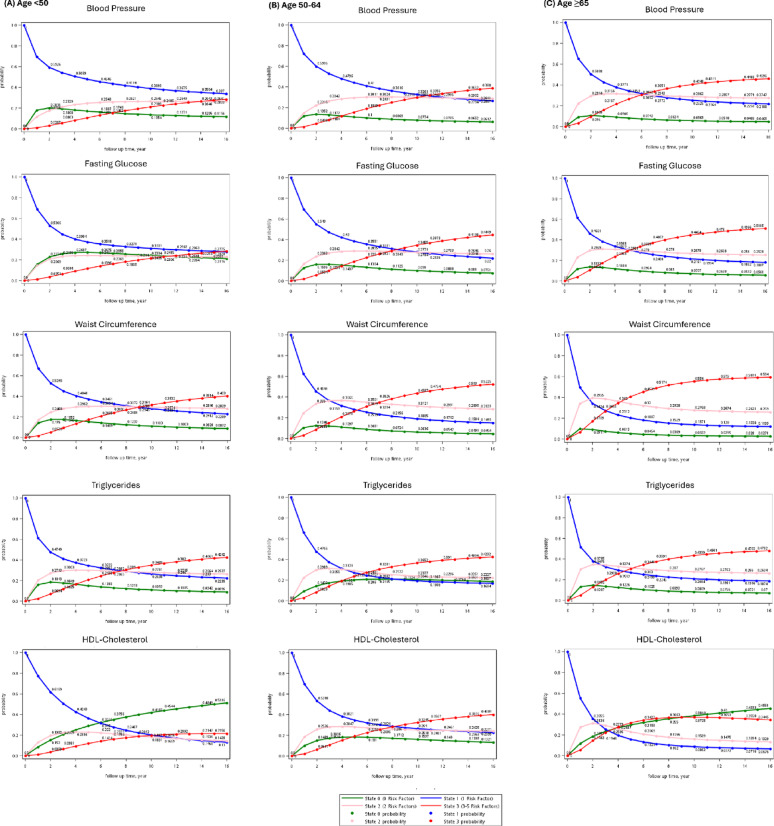
Consolidated dynamic transition probabilities from State 1 (single risk factor) over a 16-year follow-up period, stratified by age group. The panels display the probability trajectories starting from five specific initial metabolic components: Blood Pressure (BP), Fasting Glucose (IFG), Waist Circumference, Triglycerides (TG), and HDL-C. (**A**) Individuals aged < 50 years; (**B**) Individuals aged 50–64 years; (**C**) Individuals aged ≥ 65 years. The curves represent the probability of occupancy in each state over time: the **blue line** indicates remaining in the initial state (State 1); the **green line** indicates recovery to a risk-free state (State 0); the **pink line** indicates progression to two risk factors (State 2); and the **red line** indicates progression to Metabolic Syndrome (State 3, ≥ 3 risk factors). Note the varying rates of progression to State 3 (**red line**) depending on the initial component and age group

**Fig. 4:**
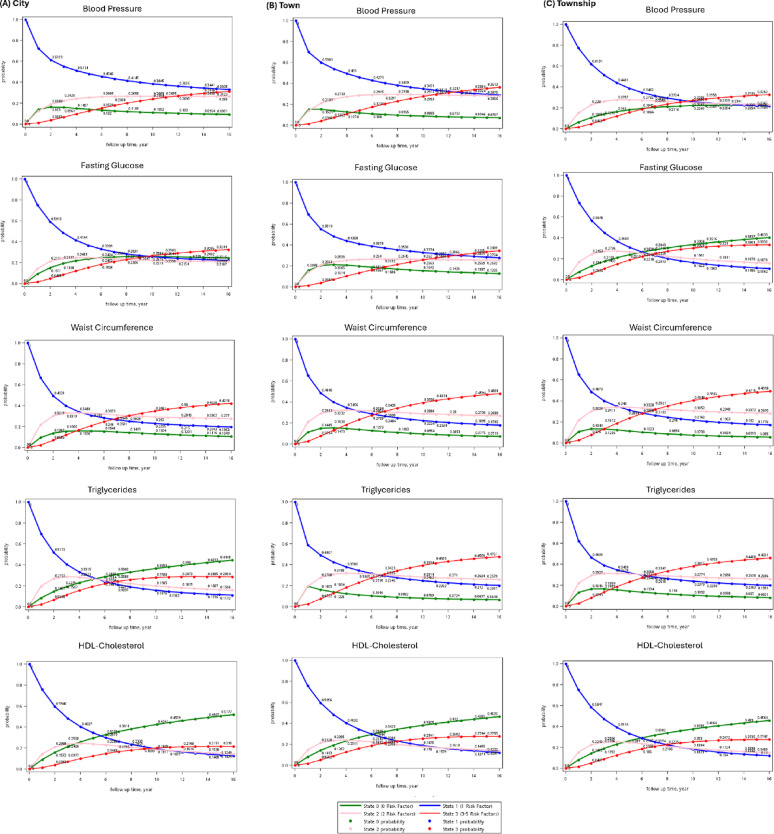
Consolidated dynamic transition probabilities from State 1 (single risk factor) over a 16-year follow-up period, stratified by residential area. The panels display the probability trajectories starting from five specific initial metabolic components: Blood Pressure (BP), Fasting Glucose (IFG), Waist Circumference, Triglycerides (TG), and HDL-C. (**A**) City, (**B**) Town, and (**C**) Township. The curves represent the probability of occupancy in each state over time: the **blue line** indicates remaining in the initial state (State 1); the **green line** indicates recovery to a risk-free state (State 0); the **pink line** indicates progression to two risk factors (State 2); and the **red line** indicates progression to Metabolic Syndrome (State 3, ≥ 3 risk factors). Note the varying rates of progression to State 3 (**red line**) depending on the initial component and urbanization level

This study shows the state occupancy probability-the likelihood of being in each state over time-over a 16-year period following baseline State 1, stratified by gender, age, place of residence, and initial anomalous component (Figs. 2, 3 and 4). According to Fig. 2, the probability of females reaching State 3 significantly increased when waist circumference or TGs levels were the initial abnormalities. The risk level of this condition exceeds that of HDL and IFG, which is consistent with the greater short-term reversibility of IFG. The male risk curve follows the same general pattern as that of females but shows a more gradual progression, which means that females experience metabolic issues at a faster rate. Figure 3 shows the age-related differences. The status shift curve shows a more significant increase in the upward direction for people aged ≥ 50 years, indicating that middle-aged and older groups experience faster metabolic deterioration. Figure 4 illustrates the regional differences in the progression to State 3. Compared to urban residents, rural/township residents who initially present with abnormal WC or TG levels are at a higher risk of developing MetS. Urban residents showed the slowest progression of initial abnormal high-density lipoprotein levels but the most significant short-term recovery.

## Discussion

In this study, we employed a continuous-time multistate Markov model to characterize the dynamic transition rates of metabolic syndrome (MetS) among middle-aged and older adults in Taiwan. This analysis utilized large-scale community-based longitudinal data for the first time and examined the heterogeneous effects of initial anomaly type, sex, age, and region on the progression of MetS. These findings indicate that the progression of metabolic abnormalities is characterized by a directional and staged pattern of accumulation, with the risk distribution varying according to individual characteristics, which has significant implications for prevention strategies and health policies.

### Asymmetry between progression and recovery rates: directional features of MetS

The findings demonstrate that the instantaneous rate of progression from a healthy or mildly abnormal state to a more advanced state (e.g., State 0→1, 1→2, 2→3) is significantly higher than the corresponding reversal rates (e.g., 3→2, 2→1, 1→0), reflecting the characteristic pattern of “fast progression and slow recovery.” This phenomenon aligns with the physiological characteristics of MetS as a chronic disease, in which the metabolic burden gradually accumulates and recovery becomes increasingly difficult [18]. Compared with Asian and European studies, the progression rates observed in this study were generally higher. For example, a Chinese longitudinal analysis using a Markov model categorized metabolic conditions into Fair Metabolic Disorder (FMD), Moderate Metabolic Disorder (MMD), and Severe Metabolic Disorder (SMD), which roughly correspond to the early, intermediate, and advanced stages of metabolic risk accumulation, respectively. The reported instantaneous transition rates from FMD to MMD, MMD to SMD, and SMD to MetS were 0.10, 0.08, and 0.10 per year, respectively [18], whereas a Spanish study reported an annual incidence rate of 38 cases per 1,000 person-years (3.8% [5]. It is important to note that the Spanish study reported incidence density, whereas our model estimates instantaneous transition intensities. Although these metrics are methodologically distinct, in the present study, the rate of State 2→3 was 0.198 per year, which was higher than the rates reported in prior studies, likely because our sample consisted predominantly of middle-aged and older adults with a higher prevalence of lifestyle risk factors (e.g., obesity and insufficient exercise) [18]. However, it is important to note that these comparisons are only approximate. Differences in MetS definitions (e.g., NCEP ATP III vs. IDF), state classification structures (3-state vs. 4-state models), and baseline population characteristics may limit the direct comparability of absolute transition intensities across studies.

The recovery rates remained low, but State 1→0 and State 2→1 reversal intensities maintained their annual rates of 0.111 and 0.181, respectively, which showed that early symptoms could improve through interventions. A previous study showed that MetS recovery improves when individuals start exercising, quit smoking, achieve weight loss, and adopt healthier dietary habits [18]. The Taiwanese healthcare system has initiated community health programs and chronic disease integrated care initiatives in recent years, which could have resulted in better recovery for patients with initial MetS symptoms [13]. Regarding long-term projections, we observed that the probability of progressing to State 3 (MetS) plateaued below 40%, whereas recovery rates from State 1 were relatively high. These patterns likely reflect the ‘healthy volunteer effect’ inherent in voluntary community screening programs, where participants are typically more health-conscious than the general clinical population. Consequently, individuals with severe or established complications (State 3) may be underrepresented as they seek hospital-based care, while those with early-stage abnormalities (State 1) remain highly responsive to lifestyle interventions.

### Initial anomaly type and progression variability

The study found that TG and WC anomalies showed the fastest progression from State 1 toward MetS, whereas IFG showed the slowest progression. Markov model simulations from previous research showed that young adults with initial WC abnormalities developed MetS at the highest rate (men: 24.6%; women: 27.6%) during the 15-year study, whereas those with IFG had the lowest risk (men: 1.31%; women: 0.64%) [9]. This study showed that TG and WC abnormalities showed fast progression but also responded well to lifestyle interventions, making them suitable for intervention programs. A systematic review showed that TG and WC are important indicators for lifestyle improvement, with TG levels decreasing by 12 mg/dL and WC measurements decreasing by 2.7 cm [19]. Previous research has identified waist circumference as the MetS component with the strongest predictive value [1]. This study shows that women between middle and older ages face distinct risk levels, as the combination of sex and creates different metabolic risk profiles. The risk of developing metabolic syndrome increases in postmenopausal women due to decreased estrogen levels, greater body fat accumulation, and worsening insulin resistance [12, 23].

### Bidirectional effects of age on the progression and recovery of metabolic syndrome

This study showed that the accumulation rate of MetS abnormalities accelerated with age, whereas the likelihood of recovery declined. A Spanish study reported that the risk of MetS was 5.56 times higher in middle-aged individuals than in younger individuals [5], and other studies have shown that middle-aged individuals have greater difficulty recovering from simple obesity than younger individuals [20].

A cross-sectional study showed that waist circumference growth represents the primary MetS feature, affecting adults, including younger populations. Young adults aged 18–28 years show the highest occurrence of MetS components through a combination of increased waist circumference, reduced HDL, and elevated TG [6].

### Influence of place of residence on the progression of metabolic syndrome

This study showed that residential settings are associated with the pace of metabolic progression. Compared with their urban counterparts, rural residents with TGs or WC as the initial abnormal components reached State 3 more rapidly. These findings likely reflect differences in the population structure, resource distribution, and health behaviors. Studies conducted in China and Africa have shown that rural populations maintain physical activity levels, but their MetS condition worsens because of elevated salt consumption, obesity prevalence, and limited healthcare services [3]. Research has revealed that MetS affects 30.2% of rural women in Shaanxi Province, China, at a higher rate than the 24.4% reported in urban women. After adjusting for socioeconomic factors, rural residence remained associated with a 27.6% higher MetS risk [22]. Early detection of abnormalities in TG, WC, blood pressure, and blood glucose levels requires better screening methods and health education programs in rural areas, together with enhanced access to primary medical care.

This study used a large population-based community screening cohort that included people from both urban and rural areas of central Taiwan to make our results more applicable to different settings. The core progression pathways showed consistent results with international studies, supporting the external validity of our results.

### Public health recommendations and practical implications

The research shows that MetS develops through multiple stages while revealing distinct risk factors that vary between sex, age, and residential area. **Our model identified elevated triglyceride (TG) levels and increased waist circumference (WC) as the earliest warning indicators associated with the fastest progression of MetS. Furthermore**,** disease progression accelerates noticeably among middle-aged and older women living in rural or township areas**,** indicating the need for immediate**,** targeted intervention approaches.**

Policy formulation requires targeted intervention strategies that address both demographic and sex-specific differences and regional profiles with elevated risk levels. Crucially, our mean sojourn time analysis revealed that individuals remained in the single-risk-factor state (State 1) for an average of 3.07 years and in the two-risk-factor state (State 2) for 2.79 years. Consequently, community health programs should move beyond generic advice and implement **targeted follow-up screenings every 2–3 years** for individuals with early-stage metabolic abnormalities. This timeframe is critical for capturing the ‘window of opportunity’ for lifestyle interventions before the condition progresses to established Metabolic Syndrome Regarding regional strategies, rural and township primary care facilities should prioritize resource allocation toward lipid management and weight control programs, as our data (Fig. 4) indicate that WC and TG are the primary drivers of progression in these areas. Although recovery rates were higher for urban residents, maintenance strategies are essential. Finally, given the steeper progression trajectories observed in older women (Fig. 2), sex-specific protocols should be intensified for postmenopausal women to detect rapid metabolic deterioration early. These evidence-based strategies will help slow the progression of metabolic disorders, thereby reducing the downstream burden of diabetes and cardiovascular diseases.

### Study limitations

The study benefits from its large community-based sample and long observation period; however, several limitations must be acknowledged in this regard. First, the study data included only participants who chose to join community screening programs, which might have produced a healthy volunteer bias in the results. Second, participants reported their daily activities through self-reporting; thus, their responses may have included recall errors and social desirability bias. Third, the Markov model in this study depends on present conditions while disregarding past exposures (the memoryless property), which simplifies a complex biological reality. Furthermore, we assumed a stepwise transition pattern (State 0 ↔ 1 ↔ 2 ↔3). While some studies (e.g., [9]) utilized complex 8-state models to capture specific component combinations, we opted for a parsimonious count-based structure to prioritize clinical interpretability and model convergence. While this stepwise structure aligns with the continuous-time premise that transitions occur one step at a time on an instantaneous scale, it does not explicitly model direct ‘jumps’ between non-adjacent states (e.g., 0 → 2) at the level of observed visits. Given the multi-year gaps between community screenings (median 3.07 years), individuals may clinically develop multiple abnormalities within a single interval. In our framework, such rapid deteriorations are treated as a sequence of unobserved intermediate transitions. This approach prioritizes model convergence and provides a clear clinical metric for progression speed, although it acknowledges that the exact timing of multiple events within long observation intervals remains unobserved.

Fourth, the model did not include a ‘Death’ state due to the unavailability of mortality data linkage. In an aging cohort, death represents a significant competing risk. To quantify the potential impact of this omission, we performed a sensitivity analysis (detailed in Supplementary Table 2) by incorporating population-level mortality rates from the 2024 Taiwan Abridged Life Table (*q*_*x*_ = 0.00658 for age 57) [15]. The simulation revealed that while the sojourn time in State 3 was reduced from 9.10 to 8.20 years, the durations for early-stage abnormalities (States 1 and 2) remained remarkably stable, with a deviation of approximately 2.1%. This confirms that the exclusion of fatal cases did not significantly bias our primary progression estimates for the critical early stages of Metabolic Syndrome, where the window of opportunity for intervention is most prominent.

Fifth, our study population was limited to individuals aged 30 years and older. Consequently, we were unable to evaluate the early onset of metabolic risk factors in young adults (< 30 years), potentially missing the initial pathophysiological origin of the syndrome. Sixth, the study period extended into the COVID-19 pandemic (2020–2021). Pandemic-related disruptions may have influenced lifestyle behaviors and screening participation, acting as potential unmeasured confounders during the final years of follow-up.

Finally, formal external validation of the model predictions against an independent dataset was not performed in this study. While the observed transitions within our large cohort (Supplementary Table 1) provide robust internal estimates, the lack of external validation suggests that the absolute transition probabilities should be generalized with caution to populations with different baseline-risk profiles.

Future research should address these limitations by integrating mortality data and applying semi-Markov models with time-dependent covariates to refine public health intervention strategies.

## Conclusion

This study employed a multistate Markov model to study MetS development using extended community screening data that monitored MetS progression from the first appearance of any abnormality. The findings indicated that waist circumference and triglyceride levels experienced the most rapid rate of change, whereas HDL-C levels showed the slowest progression among all components. The study showed that women over 40 years of age, older adults, and rural residents developed a more rapid progression of metabolic abnormalities and showed lower probabilities of recovery. Healthcare providers should identify triglyceride and waist circumference abnormalities at their onset, as these conditions require prompt intervention. The addition of time-dependent elements to future research will improve both the prediction and prevention capabilities of MetS.

## Supplementary Information

Supplementary Material 1.

## Data Availability

The Changhua County Public Health Bureau maintains ownership of the datasets, which remain inaccessible to the public. The data underwent complete de-identification before analysis; however, sharing requires authorization from the data owner. The corresponding author maintains access to the datasets but requires permission from the Changhua County Public Health Bureau to share them with the public.
