# Supplementary material for: Multistate markov model analysis of metabolic syndrome progression in a community-based longitudinal cohort study
Source: Diabetol Metab Syndr. 2026 Apr 28;18:136. doi: 10.1186/s13098-026-02146-8 (PMC13270771; doi:10.1186/s13098-026-02146-8)
Supplement: Supplementary file 1 — Supplementary Material 1. [file 13098_2026_2146_MOESM1_ESM.docx]

**Supplementary Materials**

| **Supplementary Table 1. Detailed outputs of the multistate Markov model** | | | | | | | | | | | | | | |
| --- | --- | --- | --- | --- | --- | --- | --- | --- | --- | --- | --- | --- | --- | --- |
| **(A) Matrix of Observed Transitions (Counts)** | | | | | | | | | | | | | | |
| **Baseline State** | | **Total N** | | | **Follow-up  State 0 n (%)** | | | **Follow-up State 1 n (%)** | | | **Follow-up State 2 n (%)** | | | **Follow-up  State 3 n (%)** |
| State 0 (No abnormalities) | | 14,519 | | | 7,279 (50.1) | | | 4,717 (32.5) | | | 1,792 (12.3) | | | 731 (5.0) |
| State 1 (1 abnormality) | | 17,327 | | | 3,186 (18.4) | | | 7,109 (41.0) | | | 4,567 (26.4) | | | 2,465 (14.2) |
| State 2 (2 abnormalities) | | 14,374 | | | 744 (5.2) | | | 3,187 (22.2) | | | 5,506 (38.3) | | | 4,937 (34.4) |
| State 3 (MetS) | | 16,894 | | | 181 (1.1) | | | 1,158 (6.9) | | | 3,296 (19.5) | | | 12,259 (72.6) |
| **(B) Estimated Annual Transition Intensities (q_ij_)** | | | | | | | | | | | |  |  |  |
| **Transition** | **Intensity (q_ij_​)** | | | **SE** | | **95% Confidence Interval** | | | **p-value** | | |  |  |  |
| **Progression** |  | | |  | |  | | |  | | |  |  |  |
| State 0 → 1 | 0.200 | | | 0.003 | | 0.195 – 0.206 | | | <0.0001 | | |  |  |  |
| State 1 → 2 | 0.216 | | | 0.003 | | 0.210 – 0.222 | | | <0.0001 | | |  |  |  |
| State 2 → 3 | 0.195 | | | 0.003 | | 0.190 – 0.200 | | | <0.0001 | | |  |  |  |
| **Regression (Recovery)** |  | | |  | |  | | |  | | |  |  |  |
| State 1 → 0 | 0.110 | | | 0.003 | | 0.105 – 0.115 | | | <0.0001 | | |  |  |  |
| State 2 → 1 | 0.163 | | | 0.004 | | 0.155 – 0.172 | | | <0.0001 | | |  |  |  |
| State 3 → 2 | 0.100 | | | 0.002 | | 0.096 – 0.105 | | | <0.0001 | | |  |  |  |
| **(C) Expected Mean Sojourn Times** | | | | | | | | | | | | |  |  |
| **Metabolic State** | | | **Mean Sojourn Time (Years)** | | | | **SE** | | | **95% Confidence Interval** | | |  |  |
| State 0 (No abnormalities) | | | 4.99 | | | | 0.071 | | | 4.85 – 5.13 | | |  |  |
| State 1 (1 abnormality) | | | 3.07 | | | | 0.040 | | | 2.99 – 3.15 | | |  |  |
| State 2 (2 abnormalities) | | | 2.79 | | | | 0.037 | | | 2.72 – 2.86 | | |  |  |
| State 3 (Metabolic Syndrome) | | | 9.10 | | | | 0.154 | | | 8.80 – 9.41 | | |  |  |

**Note:** This supplementary table presents detailed outputs of the continuous-time multistate Markov model. **(A)** The matrix shows the raw counts (n) and row percentages (%) of observed transitions between the baseline (*t*) and subsequent screening visits (*t*+1). **(B)** Transition intensities (*q_ij_*) represent the estimated instantaneous risk of moving between states per year. **(C)** The mean sojourn time represents the average duration (in years) that an individual is expected to remain in a specific state before transitioning to any other state, calculated as -1/q_ii_. **Abbreviations:** MetS = Metabolic Syndrome; SE = Standard Error; CI = Confidence Interval. **State Definitions:** State 0 = No abnormalities; State 1 = One abnormality; State 2 = Two abnormalities; State 3 = Three or more abnormalities (Metabolic Syndrome).

**Supplementary Table 2. Sensitivity Analysis of Mean Sojourn Times Incorporating Mortality as a Competing Risk**

| **Metabolic State** | **Original 4-State Model (Years)** | **Adjusted 5-State Model (Years)** | **Deviation (%)** |
| --- | --- | --- | --- |
| **State 0 (Healthy)** | 4.99 | 4.83 | -3.20% |
| **State 1 (1 Abnormality)** | 3.07 | 3.01 | -1.90% |
| **State 2 (2 Abnormalities)** | 2.79 | 2.74 | -1.80% |
| **State 3 (Metabolic Syndrome)** | 9.10 | 8.20 | -9.90% |

**Note:** The Adjusted 5-State Model incorporates an absorbing "Death" state to account for mortality as a competing risk. Transition intensities to death were fixed based on the 2024 Taiwan Abridged Life Table(Ministry of the Interior, 2024), using the annual probability of death for age 57 (*q_x_* = 0.00658). For State 3 (Metabolic Syndrome), an elevated mortality risk factor (1.8-fold) was assumed (*q_x_* ≈ 0.012) to reflect clinical reality. The minimal deviations in early stages (States 1 and 2) demonstrate the robustness of the primary findings regarding the window of opportunity for intervention.


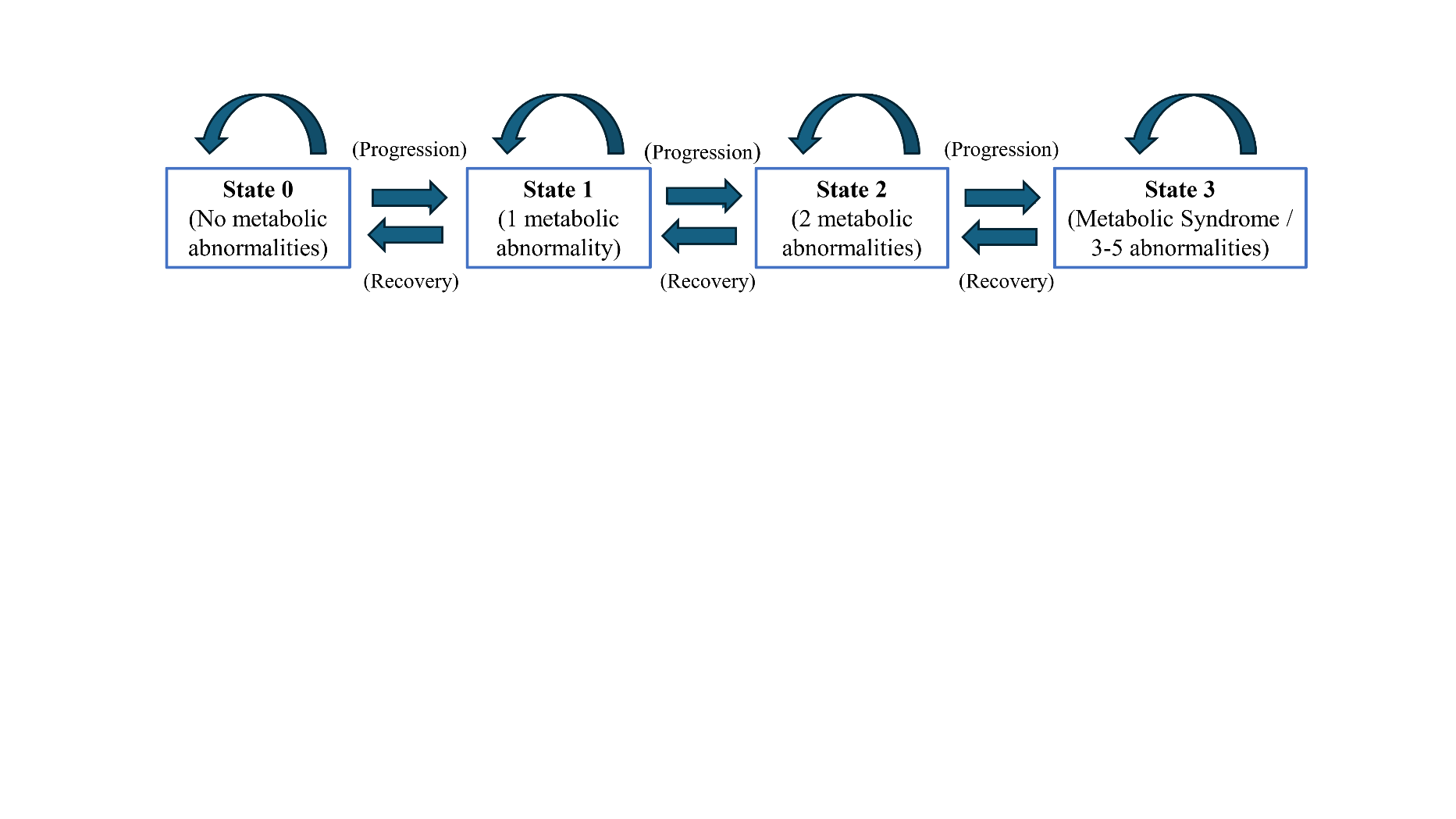


**Supplementary Figure 1. Structure of the continuous-time multistate Markov model for the progression of metabolic syndrome.** The model consists of four states based on the number of metabolic abnormalities: **State 0** (no metabolic abnormalities), **State 1** (one metabolic abnormality), **State 2** (two metabolic abnormalities), and **State 3** (Metabolic Syndrome / 3-5 abnormalities). Arrows indicate the allowable instantaneous transitions: **progression** (moving to a higher state) and **recovery** (moving to a lower state). Additionally, circular self-loop arrows on each state indicate the probability of individuals remaining in their current metabolic state. The model assumes a stepwise process in which individuals must pass through intermediate states.
